# Recovery of Bioactive Compounds from Jaboticaba Peels and Application into Zein Ultrafine Fibers Produced by Electrospinning

**DOI:** 10.3390/polym12122916

**Published:** 2020-12-05

**Authors:** Luisa Bataglin Avila, Milena Ramos Vaz Fontes, Elessandra da Rosa Zavareze, Caroline Costa Moraes, Marcilio Machado Morais, Gabriela Silveira da Rosa

**Affiliations:** 1 Engineering Graduate Program, Federal University of Pampa, 1650 Maria Anunciação Gomes de Godoy Avenue, 96413-172 Bagé, Brazil; luisabataglinavila@gmail.com; 2 Department of Agroindustrial Science and Technology, Federal University of Pelotas, 96010-900 Pelotas, Brazil; milenarvf@gmail.com (M.R.V.F.); elessandra.zavareze@ufpel.edu.br (E.d.R.Z.); 3 Graduate Program in Science and Engineering of Materials, Federal University of Pampa, 1650 Maria Anunciação Gomes de Godoy Avenue, 96413-172 Bagé, Brazil; caroline.moraes@unipampa.edu.br; 4 Chemical Engineering, Federal University of Pampa, 1650 Maria Anunciação Gomes Godoy Avenue, 96413-172 Bagé, Brazil; marciliomorais@unipampa.edu.br

**Keywords:** phenolic compounds, antioxidant, antimicrobial, active packaging

## Abstract

This work focused on the recovery bioactive compounds from jaboticaba peels and to develop ultrafine fibers from zein incorporated with the jaboticaba extract by electrospinning technique. Jaboticaba peel extracts (JPE) were obtained by maceration according a central composite rotational design (CCDR) and characterized with respect to total phenolic content (TP), antioxidant activity (AA) and total anthocyanin (TA). The optimal condition for the extraction was obtained using a desirability function in order to maximize the presence of bioactive compounds. Under these conditions the amount of cyanidin-3-glucoside (Cn-3-Glu) and the antimicrobial inhibition (AI) of *E. coli* were evaluated. Ultrafine fibers were obtained by electrospinning technique using zein in an aqueous ethanol as solvent and freeze-dried JPE at different concentrations (1.7% and 3.3%) to produce a composite membrane. The apparent viscosity and electrical conductivity of the polymer solutions, as well as the morphology, thermal stability and functional groups of the ultrafine fibers, were evaluated. The optimal conditions for extraction were 88 °C and pH 1. Under these conditions, a high amount of Cn-3-Glu was obtained (718.12 mg 100 g^−1^), along with 22.2% antimicrobial inhibition against *E. coli*. The addition of JPE into composite membranes did not affect the morphology of fibers, which presented a homogeneous and continuous format. Therefore, fibers containing JPE showed interesting characteristics for the food packaging industry.

## 1. Introduction

Jaboticaba is a native fruit of Brazil which is a rich source of anthocyanins. The variety *Plinia cauliflora* (DC) Berg belongs to the Myrtaceae family and is one of the most widely cultivated [1,2]. This fruit has a white pulp and a dark peel that is attributed to a large concentration of anthocyanins, especially Cn-3-Glu [1,3].

In addition, jaboticaba peels have a high antioxidant and antimicrobial capacity, which, together with phenolic compounds, have potential uses in the pharmaceutical and food industries. Although jaboticaba is appreciated, and despite the large production capacity of the plant (approximately 200 kg fruits per year per adult plant), it still faces obstacles in commercialization due to its perishability [4,5,6]. The berry is normally used for jelly, jam, liqueur and candy; however, the peel is not consumed and is considered to be residue. In this context, the use of jaboticaba peels is a sustainable alternative for extracting bioactive compounds, as even the polyphenols are mainly concentrated in the peel [7,8,9].

Many authors have reported the importance of studying extraction conditions and procedures, as this is an important factor affecting the phenolic compounds. Barros et al. [10] studied the effect of the acid type and changes in pH, the recovery of bioactive compounds and the antioxidant capacity of jaboticaba peels, and were able to see that the type of acid affects the recovery of anthocyanins. Ghomari et al. [11], in their study on the extraction of phenolic compounds from olive leaves, evaluated the effect of parameters such as solvent type, pH, temperature and extraction methods, such as maceration with a single step, sonication and maceration in two steps with different solvents, and proved that the extraction of the maceration in two steps, first with ethanol as a solvent and then with distilled water, was more efficient at recovering these compounds than the other tested methods. Alara et al. [12] evaluated the effect of some parameters on recovery of bioactive compounds from *Phaleria macrocarpa* fruit peel, such as time and temperature of extraction, and verified that when increasing all the parameters, the recovery of these compounds also increased.

Although there are other extraction methods, such as ultrasound-assisted, microwave-assisted and supercritical fluid, the conventional technique, maceration, is the main method applied at industrial scale for polyphenols recovery from natural materials [13,14,15,16].

Considering that jaboticaba peels are a promising source of bioactive compounds, the extracts obtained have the potential to be used as natural additives in packaging, for example. Thus, it is possible to develop new materials, such as food packaging, with active and intelligent properties based on the presence of phenolic compounds and anthocyanins in the extracts with antioxidant and antimicrobial capacity. The electrospinning technique is a simple, versatile and promising method for producing submicron fibers because of advantages such as extremely high surface area, light weight, and small diameter [17,18,19].

Some recent studies have reported the incorporation of bioactive compounds in fibers produced by electrospinning. Da Silva et al. [20] produced an ultrafine fiber with açai extracts for use as pH sensors. In another study, Wang et al. [21] developed an antimicrobial ultrafine fiber incorporating anthocyanin-rich red raspberry extract. Antunes et al. [22] studied the incorporation of eucalyptus essential oil into ultrafine zein fibers, producing a material with antimicrobial characteristics. Krumreich et al. [23] studied avocado oil encapsulated in ultrafine zein fibers to produce a material with antioxidant activity based on the carotenoids present in the oil.

The aim of this study was to extract the bioactive compounds from jaboticaba peels by the maceration method, evaluating its chemical composition and antimicrobial activity. In addition, a zein ultrafine fiber incorporated with jaboticaba extract was produced by electrospinning technique.

## 2. Materials and Methods

### 2.1. Reagents

Zein from maize (97% purity, CAS 9010-66-6), ethanol (99.9% purity, CAS 64-17-5), 2,2-diphenyl-1-picrylhydrazyl (DPPH), Folin Ciocalteu’s reagent, methanol, anhydrous (99.8%), sodium carbonate (99.5%) and gallic acid (97.5–102.5% titration) were of analytical grade. Water, acetonitrile, formic acid, Cn-3-Glu were of HPLC grade. For the antimicrobial analysis, Nutrient and Mueller-Hinton broth were used. All reagents were purchased from Himedia (Mumbai, India). The bacteria strain used in the antimicrobial experiment was *E. coli* ATCC 11229 supplied by Fiocruz–Oswaldo Cruz Foundation, Rio de Janeiro, Brazil.

### 2.2. Sample Preparation

The jaboticaba fruits (*Plinia cauliflora*) were collected from a private residence located at São José do Cedro, Santa Catarina, Brazil. The fruits were washed in running water and manually shelled. The peels were sanitized with a commercial solution of 2% sodium hypochlorite and rinsed in sterilized distilled water, then stored at −18 °C. The skins were dried in a freeze-dryer (Terroni, LS3000, São Carlos, Brazil) at −50 °C for 48 h and stored in vacuum sealed bags. The samples were reduced to a fine powder by an analytical mill (IKA, A11, Darmstadt, Germany) and sieved (Metallic mesh size 60, Metallurgical Industry Bertel, Caieiras, Brazil) to select particles with less than 0.272 mm.

### 2.3. Extraction Procedures

The bioactive compounds from jaboticaba peels were extracted by maceration technique. Extractions were carried out with addition of distilled water in a ratio of 1:100 (ground material:water). The pH of the solvent was adjusted using solutions of hydrochloric acid and sodium hydroxide, both at 0.1 M. After extraction process, the extract containing the solvent and peels powder mixture was subjected to vacuum filtration using Whatman 4 filter paper (Fisher Scientific, Hampton, VA, USA). For maceration extraction the samples were blended with water and stirred (SOLABSL-157/30, Piracicaba, Brazil), for 1 h. The level of agitation maintained a high degree of turbulence in the medium. The extraction study was based on a central composite rotational design (CCDR) with three replicates at the central point. The independent variables were temperature and pH (Table 1), and the dependent variables were total phenolic content, antioxidant activity and total anthocyanin.

Experimental data, referring to the experimental design, were analyzed using statistical software Statistica^®^, version 10.0 (SAS Institute, Cary, NC, USA) and the statistical significance was assessed through an analysis of variance (ANOVA). The dependent variables, which presented the statistical significance of the model, were optimized using the desirable function approach, while more than one response variable was considered the objective function simultaneously. In this case, the objective was to maximize the extraction of bioactive compounds.

### 2.4. Total Phenolic (TP) Quantification

The TP was quantified by a modified Folin–Ciocalteau method [24]. First, 0.5 mL of extracts was added to 10 mL of distilled water and 1 mL of Folin–Ciocalteau. After 5 min, 8 mL of 7.5% (*w*/*v*) aqueous solution of sodium carbonate were added. After 2 h at room temperature and in the dark, the absorbance was measured at 765 nm (UV 755B, EQUILAM, Diadema, Brazil). Gallic acid (50 to 1000 mg L^−1^) was used to build a calibration curve. Thus, the results were expressed as milligrams of gallic acid equivalent (GAE) per gram of dry matter. All measurements were made in triplicate.

### 2.5. Antioxidant Activity Quantification (AA)

The AA was determined by DPPH method [25]. First, 3 mL of methanolic DPPH solution (6 × 10^−5^ M) was mixed with 100 μL of the extract. After 30 min at room temperature and in darkness, the absorbance was read in spectrophotometer at 517 nm. The analysis was conducted in triplicate and the results were expressed in percentage of free radicals scavenged by DPPH radical.

### 2.6. Total Anthocyanin (TA)

Total Anthocyanin content was quantified through the spectrophotometer method. For the determination, the absorbance of the extract solution was measured at a wavelength of 520 nm, based on absorbance of Cn-3-Glu, which is the major anthocyanin in jaboticaba peels, with the spectrophotometer (Ultraspec1000, Amersham Pharmacia Biotech, Chiltern, UK). The measured concentration of anthocyanin in the jaboticaba peels was calculated using a standard curve with concentrations ranging from 5 to 100 mg L^−1^, and the results were expressed as mg of Cn-3-Glu equivalent per 100 g of dry matter.

### 2.7. Cn-3-Glu Quantification

Quantification of the amount of Cn-3-Glu in the extract was determined using the extract obtained under the optimized condition. An Agilent 1260 Infinity Series (Santa Clara, CA, USA), high performance liquid chromatography (HPLC) instrument equipped with a variable wavelength detector (VWD) was used. An isocratic elution method was used for the analysis. The separation was conducted at 30 °C using a reversed phase Discovery column (Supelco, Bellefonte, PA, USA) RP C18 (5 μm, 25 cm × 4.6 mm). The injection volume was set at 20 μL, and the flow-rate of the mobile phase was 1 mL min^−1^. The mobile phase was made up of water/acetonitrile/formic acid (80/10/10 *v*/*v*/*v*). The extracts obtained from maceration were filtered through a 0.45 mm syringe filter and directly injected in the HPLC. Compounds in the samples were identified by comparing their retention time and UV spectrum with those of the reference standard. The detector was set at 530 nm, and each sample run was conducted for 10 min. The concentrations of Cn-3-Glu in the extracts were quantified using standard curve with concentrations ranging from 500 to 4000 mg L^−1^ with characteristic peak presenting retention time between 3 and 4.5 min. The extraction yields of Cn-3-Glu were expressed in mg 100 g^−1^ dry weight (d.w.).

### 2.8. Antimicrobial Inhibition (AI)

Antimicrobial inhibition was also evaluated in the extract obtained under the optimized condition. The extract was tested against gram-negative bacteria *E. coli* following the micro-dilution method adapted to that described in the standard M07–A10 of Clinical and Laboratory Standards Institute (CSLI). Therefore, 135 μL of JPE were placed in a 96–well plate along with 145 μL of sterile Mueller–Hinton broth and 20 μL of the *E. coli* culture. The plate was incubated at 35 °C for 16 h. Two absorbance readings at 630 nm wavelength (OD630) were taken, one before the incubation period (0 h) and one after the incubation period (16 h), using a microplate reader (Celer-Polaris, Belo Horizonte, Brazil). Wells without JPE and with sterile water were used as control. Percent growth inhibition was calculated by Equation (1):

AI = {1 − [(OD_ext2_ − OD_ext1_)/(OD_control1_ − OD_ext1_)]} × 100 (1)

where AI is the inhibition (%), OD_ext2_ is the optical density for the sample after the incubation period, OD_ext1_ is the optical density for the sample before the incubation period, OD_control2_ is the optical density for the control after the incubation period, and OD_control1_ is the optical density for the control before the incubation period. The experiment was conducted in triplicate.

### 2.9. Preparation of Zein Polymeric Solutions with JPE

Zein solution was prepared according to Antunes et al. [22] by dissolving 0.9 g of zein in 3 mL of 70% aqueous ethanol (*v*/*v*), and stirred for 1 h at room temperature. The zein solutions incorporated by the JPE were prepared by dissolving extract at different concentrations (1.7% and 3.3%, *w*/*v*), in 3 mL of 70% aqueous ethanol (*v*/*v*).

### 2.10. Apparent Viscosity and Electrical Conductivity of the Polymer Solutions

The apparent viscosity of the polymer solutions was evaluated using a rheometer (Brookfield RS-CPS, Middleborough, MA, USA). An RC3-50 spindle was applied at a shear rate of 5–100 s^−1^. All measurements were conducted at room temperature (25 ± 2 °C). The electrical conductivity of the solutions was determined using a conductivity meter (model HI98311, Hanna Instruments, Woonsocket, RI, USA) and expressed in μs cm^−1^. All measurements were made at room temperature (23 ± 2 °C) in triplicate. The differences between means were analyzed using the Tukey test, with a 95% confidence level.

### 2.11. Production of Zein Ultrafine Fibers by Electrospinning Technique 

The polymeric solutions were placed in a 1 mL syringe, which had a 0.7 mm diameter bore needle, and a syringe infusion pump (KD Scientific, Model 100, Holliston, MA, USA) to control the flow rate of the polymer solution at 1 mL h^−1^. The electrospinning process was conducted by connecting the positive electrode (+18 kV) from the DC power source (INSTOR, INSES-HV30, Viamão, Brazil) to the needle, while the negative electrode (−3.2 kV) to a stainless-steel collector that was covered with aluminum foil. The horizontal distance between the needle and the collector was 15 cm. In the process, the temperature was controlled at 23 ± 2 °C by an air conditioner, and the relative humidity was set at 45 ± 2% with a dehumidifier.

### 2.12. Morphology and Diameter Distribution of Ultrafine Fibers

The morphology of the ultrafine fibers was analyzed using a scanning electron microscope (SEM) (Jeol, JSM-6610LV, Peabody, MA, USA)). Samples were analyzed at an accelerating voltage of 15 kV. The average diameter and the diameter distribution of the ultrafine fibers were evaluated from the micrographs on the basis of 60 randomly selected fibers using ImageJ (National Institutes of Health).

### 2.13. Thermal Properties of Ultrafine Fibers

The thermal stability of the ultrafine fibers was evaluated using a thermogravimetric analyzer (Shimadzu, TGA 50, Kyoto, Japan). Samples (5 mg) were heated in platinum capsules in a range of 30 to 600 °C at a heating rate of 10 °C min^−1^ with a nitrogen flow of 50 mL min^−1^.

### 2.14. Functional Groups of Ultrafine Fibers

The interaction of zein and ultrafine fibers in the jaboticaba peels extract was investigated using Fourier transform infrared–attenuated total reflection (FTIR—ATR) (Shimadzu, Prestige 21, Kyoto, Japan). A Perkin–Elmer spectrometer (UATR Two), in the range of 400 cm^−1^ to 4000 cm^−1^, was used with 32 scans per spectrum and with a resolution of 4 cm^−1^. The range was chosen to include the main bands to characterization for protein (amide I and II) in addition to the functional groups referring to polyphenols [26,27]. Then, the fibers were placed directly into the sample portal of the FTIR-ATR apparatus.

## 3. Results

### 3.1. Chemical Composition and Properties of JPE

The results of CCDR for total phenolic content (TP), antioxidant activity (AA) and total anthocyanin (TA) from jaboticaba peels obtained by maceration are shown in Table 2.

The TP content ranged from 147.68 ± 2.45 to 196.63 ± 1.39 mg_GAE_ g^−1^ d.w., which is higher than what has been reported in the literature. Aqueous extract of jaboticaba peels was also produced by maceration at 100 °C and pH 6 by Lequinste et al. [28], who found 36.12 mg_GAE_ g^−1^ d.w. Hydroethanolic extract of jaboticaba peels was produced by maceration by Meira et al. [29], who obtained 47.5 mg_GAE_ g^−1^ d.w. Palozi et al. [30], using another extraction methodology (accelerated solvent extraction), obtained aqueous extract from jaboticaba peels with total phenolic of 181.42 ± 3.67 mg_GAE_ g^−1^ d.w., which was closer to the results found in the present study.

The AA of JPE was in the range of 78.61 ± 0.21% to 94.85 ± 0.09%. Similar results were observed by Meira et al. [29] when investigating the antioxidant activity of hydroethanolic extract of jaboticaba peels, who reported 86.31%. Hydroethanolic extract of jaboticaba peels through microwave-assisted extraction was obtained by Pitz et al. [31], who reported antioxidant activity of 91.01 ± 0.42%, which was similar to the results in the current study, thus promoting greater antioxidant activity. The high values found in the study of antioxidant activity in the extracts have been widely reported to be a consequence of the presence of a large amount of phenolic compounds [10]. This statement is in line with Baldin et al. [8], which reports that the highest concentrations of anthocyanins are found in the fruit peel. According to Schreiber et al. [32], phenolic compounds can be classified as a primary antioxidants which act in the inhibition of oxidative reactions. Compounds with high antioxidant activities, as demonstrated in this study, could play an important role in the food industry, especially in the context of packaging, since they could increase the food shelf life.

The values of TA ranged from 216.24 ± 4.24 to 819.32 ± 21.44 mg 100 g^−1^ d.w. Value of 404.56 ± 35.85 mg 100 g^−1^ d.w. was reported by Lequinste et al. [28] for jaboticaba peel extract using boiling water as a solvent. In this sense, Barros et al. [10] found a result, 340 mg 100 g^−1^ d.w., inside the range obtained in this study, and also concluded that acid conditions (solvent at pH 1) promoted better recovery of these compounds. Similar results were obtained by Leite-Legatti et al. [1], at 732.77 ± 22.42 mg 100 g^−1^ d.w., using acidified ethanol 95% as solvent.

Francis and Markakis (1989) [33] reported some relevant factors when it comes to anthocyanin degradation, such as the pH of the solvent (in alkaline solvent the degradation of anthocyanins is favored), temperature (the exposure of anthocyanins for long periods in high temperatures, around 100 °C, can promote their destruction), light (the anthocyanins degradation increased when exposed to light), ascorbic acid and oxygen (both promote degradation), metals, sugars and others. Therefore, it is possible to observe, in the present study, that better results were obtained when combining acid solvent and high temperature.

The highest TA content was obtained by using acid solvent and a higher temperature for extraction. These conditions (pH and temperature) have already been reported in the literature as being favorable to the extraction of bioactive compounds from plant materials. Thus, as previously stated, Barros et al. [10] reported that acidic conditions may enhance the solubility of phenolic compounds due to the rupture of the cell wall, which increases the extraction yield, and, consequently, the rate of bioactive compounds diffusion into the solvent. Regarding extraction temperature, Cassol et al. [34], when studying the extraction of phenolic compounds from *Hibiscus sabdariffa L.* using microwave-assisted extraction, reported that the same effects mentioned above could be caused by increasing of the temperature of the matrix improving the extraction yield. Akbari et al. [35], in their study on the extraction of bioactive compounds from the fruit, drew attention to the equilibrium point between temperature and extraction time in order to increase the recovery of these compounds and prevent their degradation.

**Figure 1:**
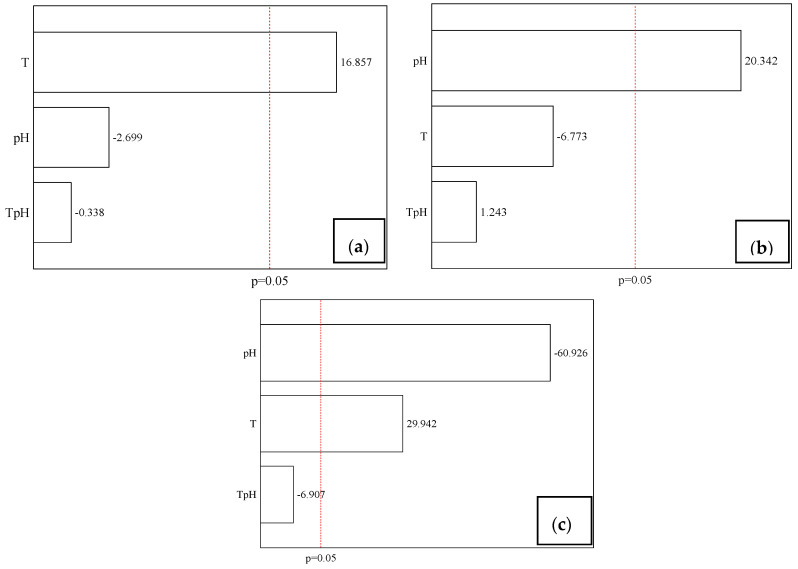
Pareto charts for TP (**a**), AA (**b**) and TA (**c**).

Thus, these results prove that the jaboticaba peels have high concentrations of phenolic compounds and anthocyanins, which have important properties such as antioxidant activity, which can be proven through analysis, which shows high antioxidant activity (94.8%). These answers make it possible to infer that jaboticaba peels have potential uses as natural additives and could add economic value to the residue, in addition to contributing to food safety, increasing the shelf life of packaged products. Figure 1 presents the influence of independent variables on TP, AA and TA, with a significance level of 95% (*p* < 0.05).

The Pareto charts show that the most pronounced effect on extraction of anthocyanins was solvent pH, which had a negative influence. On the other hand, the effect that showed the greatest influence on total phenolic extraction was temperature (T). This variable had a positive influence on both phenolic and anthocyanin content.

Thus, it is possible to infer that the pH of the solvent is inversely proportional to the anthocyanin content, that is, in acid medium the extraction of the same is more effective. These results are in agreement with what is described in the literature, where according Blackhall et al. [36], the addition of acid to the extraction solvent may increase the extraction of phenolic compounds in special anthocyanins, since in the acid medium there is cell membrane denaturation that promotes the increase of the interaction between solvent and compound and, in addition, hydrogen-free ions lead to stabilization of the anthocyanin flavylium cation form. Moreover, it is possible to observe that extraction temperature is directly proportional to the content of phenolic and anthocyanin compounds. When studying different extraction techniques of phenolic compounds from *Pinus* bark, Aspé and Fernández [37] found that extraction was more efficient when using higher temperatures. This effect can be explained by the decreased surface tension and solvent viscosity, accelerating the solubilization of analytes at this phase [38]. 

On the other hand, the major effect in antioxidant activity was the solvent’s pH, which presents a negative effect, suggesting that alkaline solvent is more efficient for obtaining antioxidant properties. This is possibly because, although the alkaline medium causes the anthocyanins degradation, some other compounds, rich in antioxidant properties, can be released. This fact is related with the composition of jaboticaba peel, which, according to Neves et al. [2], contains a large quantities of tannins, such as gallotannins and ellagitannins which, as described by Wu et al. [39] have many properties, including antioxidant activity. In this sense, Ham et al. [40] evaluated the antioxidant activity of chestnut inner shell extracts and prove that alkaline solutions were more effective in extracting specific phenolics, such as tannins. 

Second-order polynomial models were obtained to estimate the dependent responses. The statistical significance of the models was verified through ANOVA and is presented in Table 3.

The regression can be considered significant when the F_value_ is higher than the F_tabled_, and in the models for estimating the TP and TA, this criterion was fulfilled. However, the proposed model for AA was not statistically significant, and therefore cannot be considered predictive. In line with this, the determination coefficient shows the same. For models TP, AA and TA, this coefficient was 96.12%, 60.02% and 91.86%, respectively. In addition to that, the adjusted determination coefficients for TP, AA and TA were 99.84%, 99.91% and 99.98%, respectively. The predicted models are presented by Equations (2) and (3).

TP = 149.38 + 14.29 T − 2.29 pH + 12.13 T^2^ + 16.30 pH^2^ − 0.4 T pH (2)

TA = 388.96 + 81.82 T − 166.48 pH + 6.66 T^2^ + 81.66 pH^2^ − 26.69 T pH (3)

**Figure 2:**
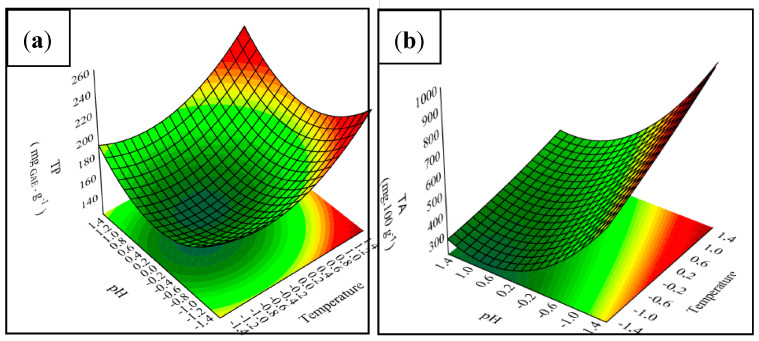
Responses surfaces for TP (**a**) and TA (**b**).

Therefore, response surfaces were used to illustrate the effect extraction temperature (T) and extraction pH on the responses that presented predictive models according to ANOVA, and are shown in Figure 2.

For the optimization of TP, it is possible to infer that high temperatures favor the extraction process. Regarding the solvent, it is clear, through Figure 2a, that the best results are achieved using solvent with both extreme pH (low and high). However, although anthocyanins belong to the group of phenolic compounds, the response surfaces have distinct curvatures. This difference is related to the solvent pH, since the alkaline medium promotes TA degradation. Nevertheless, some phenolic compounds can be extracted using an alkaline solvent; hereupon, some authors have related the application of alkaline treatment to the extraction of phenolic compounds [41,42].

**Figure 3:**
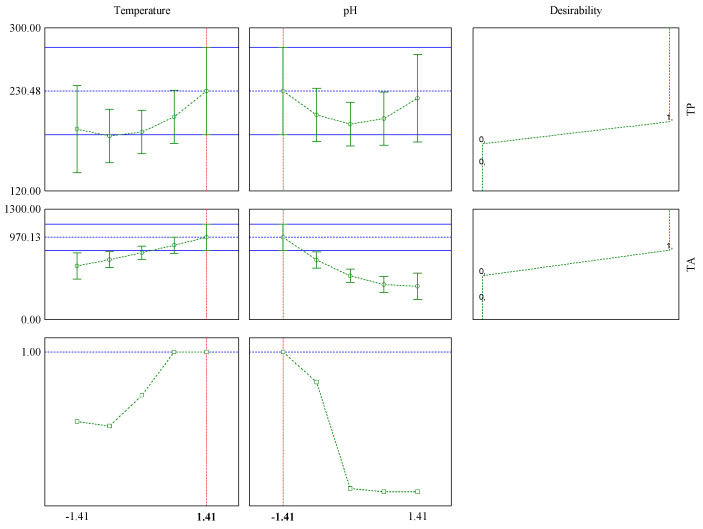
Desirability function.

Based on the statistical significance of the models, the optimal condition for extraction of the TP content and TA was obtained by the desirability function. The results are presented in Figure 3, where the optimal values of the factors are presented.

According to the purpose of the optimization—in this case, maximizing the recovery of bioactive compounds present in jaboticaba peels—individual desirabilities were chosen. In this case, the interest was only in the maximum extraction of bioactive compounds, so a convenience of 1.0 was assigned for maximum TP and TA. 

The maximum overall desirability reached the optimal point with a value of 1. According to the Harrington scale, shown in Table 4, this value is related to the quality assessment and is considered acceptable and excellent; it is also worth noting that there is no preference for improvement beyond that point.

Thus, it can be verified that the optimum condition for the total phenolic content extraction and anthocyanin extractions is the +1.41 level of temperature and the −1.41 level of pH solution, i.e., 88 °C and pH 1, respectively, predicting values of 230.48 mg_GAE_ g^−1^ for TP and 970.13 mg 100 g^−1^ for TA. Under these conditions, the real values obtained were 199.34 mg_GAE_ g^−1^ and 1458.11 mg 100 g^−1^ for TP and TA, respectively. These results show the importance of use the desirability function, because although the real values were slightly higher than predicted, the use of the optimized condition made it possible to obtain values of TP and TA higher than those obtained in the experimental design matrix.

**Figure 4:**
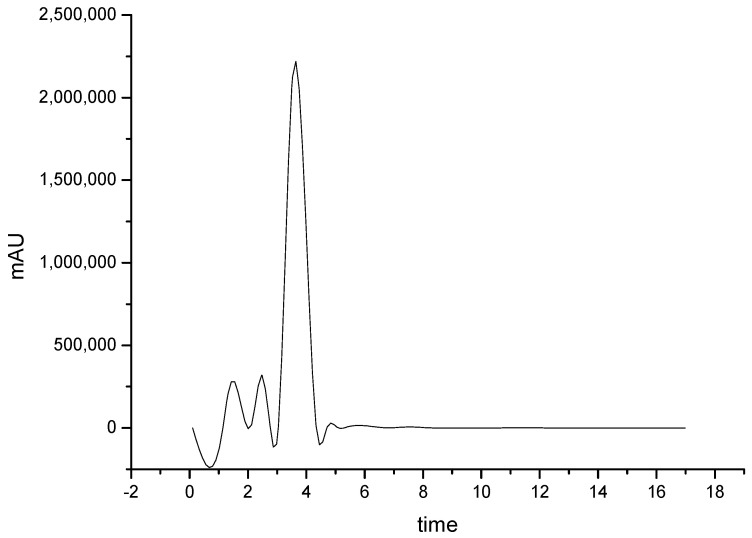
HPLC chromatogram at 530 nm of jaboticaba peels extract obtained under optimal conditions.

Figure 4 shows the chromatogram of jaboticaba peel extract obtained by the optimal condition at 530 nm.

The presence of Cn-3-Glu in jaboticaba peel extract was observed by the optimal extraction conditions (Figure 4). Under these conditions the amount of Cn-3-Glu obtained from HPLC was 718.12 mg 100 g^−1^, which corresponds to 49.25% of the TA. The results obtained were higher than those obtained by Wu et al. [39], 298 mg 100 g^−1^ for whole jaboticaba (*Myrciaria cauliflora*) fruit that used methanol as a solvent and ultrasonic extraction combined with maceration extraction. They were also higher than those obtained by Gurak et al. [44] using *Myrciaria cauliflora* species, who found a concentration of 358 mg 100 g^−1^ for the extract of jaboticaba peel in which ethanol 85% was used, acidified, as solvent. Inada et al. [4] reported a value higher than that obtained in this study: 1261 mg 100 g^−1^ for jaboticaba peel extract using methanol as solvent. These differences could be related to the type of the solvent, as well as to fruit variety and fraction of the fruit used in the extraction.

According to Naczk et al. [42], the choice of solvent is an important factor to observe, since the solvent governs the solubility of bioactive compounds from plant materials. In line with this, Mood-Esa et al. [45] evaluated the use of water and methanol 80% as solvent for recovery of bioactive compounds from hibiscus, and proved that methanol promoted higher values than water. In relation to these fractions of fruit, Inada et al. [4] reported that major concentrations Cn-3-Glu were found in the peels. In addition, Neves et al. [2] studied the chemical composition of different species of jaboticaba, including the species used in the present study (*Plinia cauliflora*), and proved the influence of fruit variety.

The antimicrobial inhibition (AI) of JPE obtained under the same conditions was tested against *E.coli* and showed 22.21% inhibition of the growth at a concentration of 50 mg mL^−1^. The results obtained were higher than those reported by Özkan et al. [46], who found that grape pomace extract at 50 mg mL^−1^ inhibited the growth of *E. coli* by 11%. The inhibition is related to the composition of JPE, and certainly to the high concentration of phenolic compounds. Hereupon, Borrás-Linares et al. [47] related that phenolic compounds from plant materials have important antimicrobial properties that normally act through destabilization of the cell wall. Thus, the rupture of cytoplasmic membrane occurs, and several mechanisms are initiated such as enzyme inactivation and protein denaturation, which retards bacterial growth and multiplication.

Therefore, the antimicrobial effect of jaboticaba extracts has been described in literature. Some authors [8,48] used another method to investigate the antimicrobial capacity of jaboticaba extract and also reported antimicrobial inhibition against *E. coli* at a concentration of 50 mg mL^−1^. According to these considerations, it is possible to infer that jaboticaba is an important source of bioactive compounds with a potential use as a natural antimicrobial additive.

### 3.2. Apparent Viscosity and Electrical Conductivity of the Polymer Solutions

Apparent viscosity and electrical conductivity of polymeric solutions with different concentrations of JPE are reported in Table 5. Similar results were reported by Niu et al. [49] and Antunes et al. [22], who found values of 192.47 ± 9.62 and 192.3 ± 11.0 μs cm^−1^, respectively, for electrical conductivity.

The electrical conductivity of the solutions increased with the increase of JPE concentration, and according the Tukey’s test, there was significant difference between the means. This effect can be attributed to the pH of the solutions, since the extracts were obtained in an acid medium (pH 1). Similar results were obtained by Son et al. [50], who studied the effect of pH on electrospinning of poly(vinyl alcohol) and concluded that in an acid medium the electrical conductivity of the polymeric solutions is greater than in a neutral medium.

According to Aydogdu et al. [51], the electrical conductivity of a solution reflects the ability of charge on a jet so it directly affects the elongation level of a jet by electrical force caused by greater mobility of ions. In general, under the same processing conditions, while higher electrical conductivity cause higher elongation of jet and fibers with smaller diameter.

Viscosity is one of the main parameters related to the formation of ultrafine fibers and the composition of the solution is strictly related to this parameter [52]. It was observed that the solution with only zein showed a higher value, with 0.39 Pa s, and the addition of the extract promoted a reduction in viscosity, probably due to the presence of water in the JPE. According to the Tukey test, the addition of the extract caused a significant difference in this parameter; however, the same was not observed with the increase of extract concentration.

### 3.3. Morphology of Ultrafine Fibers

**Figure 5:**
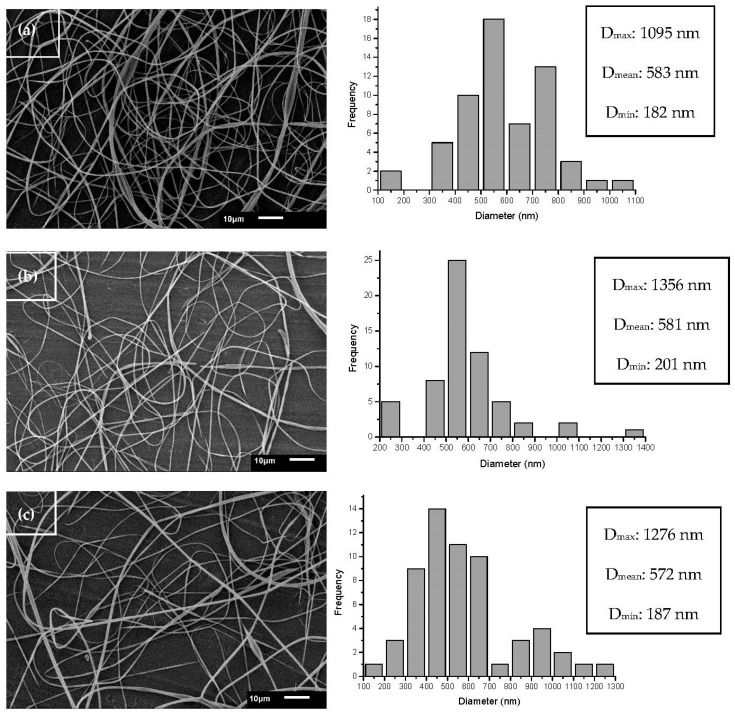
Ultrafine fiber morphology and size distribution from zein 30% (**a**), zein 30% + 1.7% JPE (**b**) and zein 30% + 3.3% JPE (**c**). JPE: jaboticaba peel extract.

The effects of zein and JPE concentrations (0%, 1.7% and 3.3%) on ultrafine fiber morphology are shown in Figure 5a–c, respectively. The conditions used in processing allowed the formation of fibers with homogeneous and continuous formats without the presence of beads. The addition of JPE did not affect the morphology of the fibers; however, the addition of JPE reduced the mean fiber diameter. The mean diameter of fibers ranged from 583 nm for zein-only fibers (Figure 5a) to 572 nm for fibers with zein and 3.3% JPE (Figure 5c). These results corroborate the results observed in electrical conductivity.

According to Kim, Lee and Kim [53], higher electrical conductivity promotes a greater mobility of ions, and consequently, with the application of an electric field they tend to be directed more easily since the overall tension in the fibers is dependent on self-repulsion from the excess charges on the jet. Therefore, as the electrical conductivity increases, the diameter of the resulting fibers and the number of beads decrease.

In this study, as expected, it was found that the diameter of the ultrafine fibers decreased with the decrease in the viscosity of the polymeric solutions, a desired characteristic that is in line with what was described in [54].

A similar mean diameter of zein fibers was reported by Dashdorj et al. [55], who found a value of 500 nm, and according Chen et al. [56], they can be classified as ultrafine fibers. Polymeric materials may have better mechanical strength, higher thermal stability, higher electrical conductivity, etc. Therefore, these materials are very promising in the food packaging industry, because they have improved mechanical and barrier properties in addition to presenting potential development of advanced structures with active and intelligent properties [57,58].

### 3.4. Thermal Properties of Ultrafine Fibers

**Figure 6:**
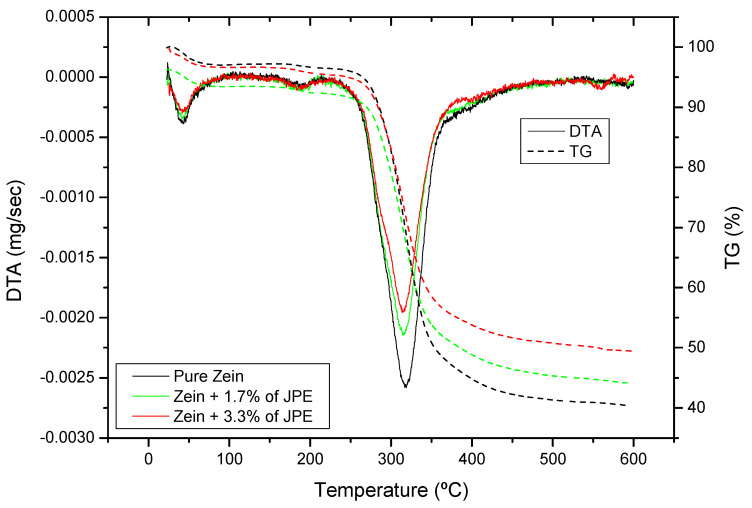
Thermogravimetric curves of zein ultrafine fibers with different concentrations of jaboticaba peel extract (JPE).

Thermogravimetric analysis was performed to evaluate the thermal stability of the constituents of ultrafine fibers. TGA and DTA curves are shown in Figure 6 of zein ultrafine fibers with different concentrations of JPE.

Because the low thermal stability of phenolic compounds makes them prone to degradation at high temperatures, their incorporation in zein fibers is desired to potentially increase their thermal stability. In line with this, the incorporation of JPE in ultrafine fibers, regardless of concentration, did not influence the initial decomposition temperature of zein ultrafine fibers. The TGA curves showed two decomposition zones. The weight loss in the first zone (below 170 °C) is usually attributed to evaporation of water and volatile components [59]. In the second zone (around 270 °C) a rapid decomposition occurs due to the degradation of zein.

The results revealed that the greatest mass loss occurred in the range 250–370 °C, which can be attributed to proteins breakdown and peptide bond cleavage [22]. The addition of JPE in the polymeric matrix improved the thermal stability of the fibers, and this factor can be observed by the residual weight of the fibers which were 41%, 45% and 50% for the pure zein fiber, fiber with 1.7% JPE and fiber with 3.3% JPE, respectively. This thermal stability may be related to the presence of phenolic compounds from JPE. In their study, Barbosa et al. [60] reported that the decomposition of phenolic compounds occurs at temperatures between 500 and 580 °C.

### 3.5. Functional Groups of Ultrafine Fibers

**Figure 7:**
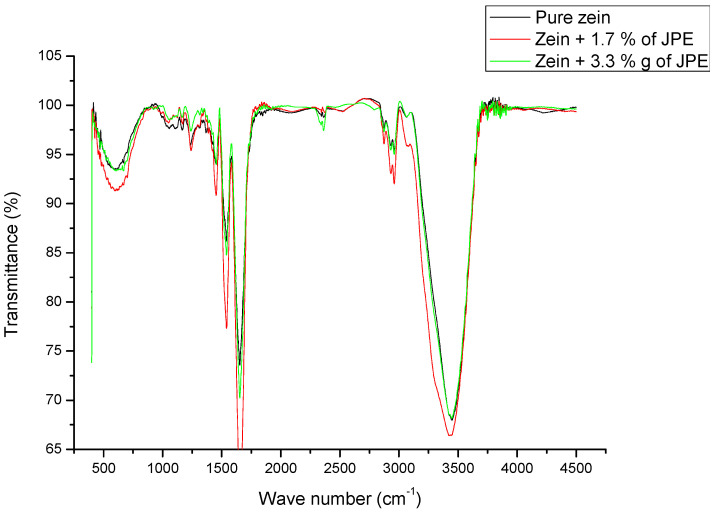
FTIR spectra zein fibers with different concentrations of jaboticaba peel extract.

The chemical interaction between zein ultrafine fibers and JPE was investigated by FTIR-ATR analysis, as presented in Figure 7.

Pure zein fiber and fibers with JPE showed amide I band identified at 1652 cm^−1^. The amide I band is related to C=O bond stretching. However, the amide II, identified at 1542 cm^−1^ for pure zein fiber and 1540 and 1539 for zein fibers with 1.7% and 3.3% JPE, respectively, is associated both to N-H bending and C-N stretching. The addition of JPE caused the 1542 cm^−1^ band of amide II to shift to 1540 cm^−1^ and 1539 cm^−1^ with the addition of JPE (1.7% and 3.3%, respectively), suggesting that the added JPE interacted with the amine group of amide II. A similar result was observed by Antunes et al. [22], who studied the interaction between zein fibers and eucalyptus essential oil.

The frequencies of amide I and II are related to the size of the α-helix structure. A lower wavenumber indicates greater structural stability, which means that there is a shift of the band to a region of lower wavenumber [61]. This fact is associated with the increased hydrogen bonding interaction occurred at the N-H group, which favors the formation of the most stable structure [62].

Bands at 1160–1030 cm^−1^ for the asymmetric stretching of the C-C and axial stretching of the C-O bond were also observed in Figure 7. These bands were due to the polysaccharides present in the zein [22,63]. 

According to some authors [26,55] the peaks observed at 2959 and 2930 cm^−1^ could be indicate the C-H stretching from CH_3_ and CH_2_ functional groups. These peaks can be derived from free fatty acids from zein, probably due to fat residue, which is common in commercial zein [26].

The spectrum for the zein fiber with 3.3% extract shows absorption bands at 1652 and 1453 cm^−1^ that can be attributed to the stretching vibration of the C=C aromatic ring. These peaks can also be attributed to the presence of polyphenols groups present in the extracts [64]. This absorption is not observed in the spectrum of fiber containing 1.7% extract. This factor can be explained by the low concentration of extract, which contains the compound, in the fiber. Moreover, the bioactive compounds present in the extract are expected to be reduced when incorporated into other products, such as ultrafine fibers, for example. Thus, a minimum concentration of the extract is required to ensure the presence of compounds such as anthocyanins.

On the other hand, band intensity differences between fibers prepared with 1.7% and 3.3% lyophilized JPE may be related to the homogeneity of the extract in the preparation of electrospinning solutions. Erdogan et al. [65], when studying the preparation of zein fibers by electrospinning with the addition of olive leaf extract, compared the chemical interaction between zein fibers incorporated with aqueous and lyophilized olive leaf extract and observed that the aqueous extract was more integrated into the zein structure than lyophilized extract.

Based on this, the addition of JPE to the zein fibers is promising for application in active food packaging, and other concentrations of the same can be tested.

## 4. Conclusions

The jaboticaba peel extracts were obtained by maceration based on a central composite rotational design and analyzed by chemical composition and antimicrobial inhibition. The experimental results showed that jaboticaba peel extracts have high total phenolic content, antioxidant activity and total anthocyanin. The desirability function predicted values, under optimal conditions, of 230.48 mg_GAE_ g^−1^ of total phenolic content and 970.13 mg 100 g^−1^ of anthocyanins. Additionally, the jaboticaba peel extract obtained under this condition showed antimicrobial activity of 22.2% against *E. coli* at an extract concentration of 50 mg L^−1^, and concentration of Cn-3-Glu of 718.12 mg 100 g^−1^. Thus, the high content of bioactive compounds with antimicrobial and antioxidant properties present in JPE suggest that it could have an important application as a natural additive in food packaging.

The electrospinning technique was used to produce zein ultrafine fibers incorporating jaboticaba (*Plinia cauliflora*) peel extract. In addition to that, the incorporation of JPE into polymeric solutions increased electrical conductivity and reduced viscosity, which allowed the formation of fibers with smaller diameters than pure zein fibers and homogeneous morphology without the presence of beads. The thermogravimetric analysis showed that incorporation with JPE did not influence the degradation temperature and the highest mass loss occurred in the range 250–370 °C, which is a good property for applications in food packaging that require thermal processing and exposure to irradiation. It was also possible to confirm functional groups of the phenolic compounds on the surface, proving the presence of JPE in the fibers.

The presence of bioactive compounds into the fibers may allow the use of extract as a natural additive to obtain packaging with active properties. Additionally, the presence of a large amount of anthocyanins could indicate that this biopigment could be used as a colorimetric pH sensor. Future studies are needed to evaluate the antimicrobial activity of these ultrafine fibers and their intelligent capacity.

## Figures and Tables

**Table 1:** Independent variables of experimental design and extraction conditions.

Variables	Levels				
	−1.41	−1	0	+1	+1.41
**T (°C)**	32	40	60	80	88
**pH**	1	2.5	6	9.5	11

**Table 2:** Experimental results according CCDR.

Run	T (°C)	pH	TP (mg_GAE_ g^−1^ d.w.)	AA (%)	TA (mg 100 g^−1^ d.w.)
1	40	2.5	160.58 ± 2.01	94.19 ± 0.30	513.8 ± 5.01
2	40	9.5	163.06 ± 1.03	94.85 ± 0.09	327.7 ± 8.52
3	80	2.5	189.05 ± 0.85	92.35 ± 0.58	749.27 ± 7.47
4	80	9.5	189.91 ± 1.11	94.13 ± 0.18	456.41 ± 13.46
5	32	6	154.93 ± 1.18	94.67 ± 0.14	265.11 ± 0.91
6	88	6	196.63 ± 1.39	90.52 ± 0.92	470.42 ± 12.56
7	60	1	191.78 ± 1.88	78.61 ± 0.21	819.32 ± 21.44
8	60	11	176.48 ± 2.12	94.70 ± 0.65	216.24 ± 4.24
9 (C)	60	6	151.07 ± 3.06	93.92 ± 0.27	383.5 ± 3.03
10 (C)	60	6	147.68 ± 2.45	94.54 ± 0.02	394.43 ± 0.70
11 (C)	60	6	185.52 ± 2.06	92.80 ± 0.13	373.97 ± 8.86

(C) Central point.

**Table 3:** Analysis of Variance (ANOVA).

	Variance Analysis	Sum of Squares	Degrees of Freedom	Mean of Squares	F_value_	F_tabled_
**TP**	Regression	3562.34	5	712.47	23.27	6.25
	Residual	122.46	4	30.62		
	Lack of fit	116.72	3	38.91	154.20	215.71
	Pure error	5.75	1	5.75		
	Total	3684.8	9			
**AA**	Regression	122.88	5	24.58	1.10	6.25
	Residual	89.10	4	22.28		
	Lack of fit	88.91	3	29.64	154.20	215.71
	Pure error	0.19	1	0.19		
	Total	211.99	9			
**TA**	Regression	306,582.8	5	61,316.56	8.93	6.25
	Residual	27,451.5	4	6862.88		
	Lack of fit	27,379.7	3	9126.57	127.11	215.71
	Pure error	71.8	1	71.8		
	Total	334,034	9			

**Table 4:** Harrington qualitative definition of the Desirability (D) scale.

Scale of D	Quality Evaluation
1.00	No preference for improvement beyond this point
1.00–0.80	Acceptable and excellent
0.80–0.63	Acceptable and good
0.63–0.40	Acceptable but poor
0.40–0.30	Borderline
0.30–0.00	Unacceptable
0.00	Completely unacceptable

Source: Tauler et al. [43].

**Table 5:** Apparent viscosity and electrical conductivity of polymeric solutions with different concentrations of jaboticaba peel extract (JPE).

Polymer Solutions	Apparent Viscosity (Pa s) *	Electrical Conductivity (μs cm^−1^)
Pure zein (30%)	0.39 ^b^ ± 0.06	235.9 ^a^ ± 2.7
Zein 30% + 1.7% JPE	0.21 ^a^ ± 0.08	249.0 ^b^ ± 1.3
Zein 30% + 3.3% JPE	0.13 ^a^ ± 0.01	256.4 ^c^ ± 1.2

* At a shear rate of 35 s^−1^; different letters in the same column show significant difference between the means in a Tukey’s test (*p* < 0.05).
